# The safety and efficacy of semaglutide in people with schizophrenia spectrum disorders: systematic review and meta-analysis of randomised controlled trials

**DOI:** 10.1192/bjo.2026.12001

**Published:** 2026-05-25

**Authors:** Mike Trott, Urska Arnautovska, Donni Johnston, Gabrielle Ritchie, Dan Siskind

**Affiliations:** Princess Alexandra Hospital Southside Clinical Unit, The University of Queensland Faculty of Medicine, Australia; Addiction and Mental Health Services, Metro South Hospital and Health Service, Australia; Queensland Centre for Mental Health Research, Wacol, Australia; Faculty of Health, Medicine and Behavioural Sciences, The University of Queensland, Australia

**Keywords:** Psychotic disorders/schizophrenia, randomised controlled trial, clinical trials, systematic review, meta-analysis

## Abstract

**Background:**

People with schizophrenia spectrum disorders (SSDs) experience high rates of obesity and metabolic dysfunction, contributing substantially to excess morbidity and mortality. Glucagon-like peptide-1 receptor agonists (GLP-1 RAs) such as semaglutide and tirzepatide have demonstrated substantial efficacy for weight and glycaemic outcomes in the general population, but evidence in people with SSDs remains limited.

**Aims:**

To synthesise all placebo-controlled, randomised controlled trials (RCTs) examining semaglutide and/or tirzepatide in people with SSDs.

**Method:**

A preregistered systematic review and meta-analysis of RCTs examining the efficacy and safety of semaglutide and/or tirzepatide in adults with SSDs was conducted. Outcomes and adverse events were pooled using random-effects meta-analysis. Certainty of evidence was assessed using the GRADE criteria.

**Results:**

Three trials (*n* = 258) met inclusion criteria, examining semaglutide dosages of 1.0–2.0 mg over 26–36 weeks. No trials examining tirzepatide were found. Semaglutide significantly reduced body weight (−11.32 kg; 95% CI −15.35 to −7.29), body mass index (−3.58 kg/m^2^; 95% CI −4.86 to −2.30), haemoglobin A1c (−0.37%; 95% CI −0.51 to −0.22) and fasting glucose (−0.54 mmol/L; 95% CI −0.94 to −0.13). In adverse event analyses, semaglutide was associated with increased risks of abdominal pain (risk ratio 2.93; 95% CI 1.13–7.60), vomiting (risk ratio 2.57; 95% CI 1.39–4.77) and constipation (risk ratio 3.23; 95% CI 1.14–9.18). There was no evidence of increased risk of serious adverse events.

**Conclusions:**

Semaglutide produces clinically meaningful improvements in weight and glycaemic outcomes in people with SSDs, with an adverse event profile consistent with known gastrointestinal effects of GLP-1 RAs in the general population. These findings support semaglutide as a promising adjunctive metabolic intervention in this population, although larger and longer trials, specifically those testing tirzepatide, are needed to better characterise heterogeneity of effects and long-term safety of these promising pharmacological treatments.

Schizophrenia spectrum disorders (SSDs), including schizophrenia and schizoaffective disorders, are among the leading causes of disability worldwide, with their rising prevalence causing increased burden over recent decades.^1,2^ People living with SSDs experience profound reductions in life expectancy, dying an average of 10–20 years earlier than the general population.^3^ The majority of these of premature deaths can be attributed to physical health comorbidities, particularly outcomes related to metabolic syndrome, such as cardiovascular disease (CVD).^3^

Weight gain is a major modifiable risk factor for CVD in SSDs, with the side-effects of antipsychotic medications playing a central role. For example, meta-analyses of individuals initiating antipsychotic treatment report rapid weight gain, with clozapine and olanzapine associated with the greatest increases.^4,5^ Further evidence indicates that switching to certain antipsychotic medications can induce additional weight gain.^6^ The burden of antipsychotic-associated weight gain goes beyond increased risks of CVD and metabolic syndrome, contributing to increased stigma,^7^ reduced treatment adherence^8^ and poorer quality of life.^9^ Behavioural approaches, including structured dietary modification and exercise programmes, have shown some efficacy for weight management in people with SSDs; however, their effects are typically modest, and implementation can be resource intensive.^10,11^ Pharmacological adjuncts have, therefore, been explored as alternative or complementary strategies. Among these, metformin has demonstrated modest efficacy in reducing antipsychotic-associated weight gain, with pooled estimates suggesting an average benefit of approximately 3 kg relative to placebo and no clear excess of adverse events.^12^ Despite these gains, the overall impact of existing behavioural and pharmacological interventions remains insufficient to address the high prevalence and severity of obesity observed in SSDs, underscoring the need for more effective therapeutic options.^13^

Glucagon-like peptide-1 receptor agonists (GLP-1 RAs) have emerged as a major advance in the management of obesity and type 2 diabetes in the general population, facilitating weight loss through a combination of glucose-dependent insulin secretion, delayed gastric emptying and centrally mediated appetite regulation.^14,15^ Early GLP-1 RAs such as exenatide and liraglutide produced clinically meaningful weight loss in the general population.^16^ An individual participant data meta-analysis of exenatide and liraglutide trials in people with schizophrenia reported modest weight loss of almost 4 kg, which is comparable to that observed with metformin, but also identified higher rates of adverse events among participants receiving these two agents.^17^ In contrast, newer GLP-1 RAs such as semaglutide and tirzepatide have demonstrated substantially greater weight reductions in the general population. For example, Wilding and colleagues reported weight loss of almost 15% versus placebo after 68 weeks of 2.4 mg semaglutide.^18^ Furthermore, studies examining tirzepatide have yielded substantial weight loss exceeding 15% across similar time frames,^19^ with comparative effectiveness studies concluding that tirzepatide is superior to semaglutide in terms of weight loss.^20^ Similarly to the large liraglutide and exenatide studies, these large semaglutide and tirzepatide studies excluded people with SSDs. Of the trials that have examined semaglutide in people with SSDs, results have mirrored and exceeded those seen in the general population, with no evidence of unwanted effects regarding medication interaction or psychosis symptoms.^21^ However, results of semaglutide and tirzepatide studies have not been systematically evaluated in SSD populations to date. The aim of this study, therefore, is to synthesise the available evidence evaluating the effectiveness and safety of semaglutide and/or tirzepatide in individuals with SSDs.

## Method

This systematic review and meta-analysis was conducted as per the Preferred Reporting of Items for Systematic Reviews and Meta-Analyses (PRISMA) criteria,^22^ and followed a publicly available protocol (PROSPERO identifier: CRD420251247162; registered 9 December 2025; data extracted 15 December 2025; no amendments were made to the protocol). The PRISMA checklist can be found in the Supplementary Material.

Searches were conducted on 6 December 2025 across PubMed, EMBASE, PsycINFO, Scopus and CENTRAL databases. The full search strategy can be found in the Supplementary Material. Duplicates were automatically excluded, with titles and abstracts screened by two independent reviewers (M.T. and K.V. (see ‘Acknowledgements’ section)). Following title and abstract screening, full-text articles were retrieved and screened by two independent researchers (U.A. and K.V.), and data extracted by two independent researchers (M.T. and K.V.) using a custom spreadsheet. Agreement between independent reviewers was 100% across all screening and extraction stages, therefore no senior arbitration was required. The reference lists of these full texts were also examined. The inclusion criteria used to identify appropriate studies were:

- population: people diagnosed with SSDs according to the ICD-x or DSM-x criteria, or via medical records – studies including mixed diagnostic populations were eligible if results for participants with SSDs were reported separately, or if the recruited sample consisted exclusively of individuals with SSDs despite broader eligibility criteria;
- intervention: semaglutide or tirzepatide at any dosage across any time frame;
- comparator: placebo control groups only;
- outcome: any outcomes;
- study design: randomised controlled trials (RCTs) only.

### Statistical analysis

Outcomes were meta-analysed when there were at least three component studies. If fewer than three studies reported similar outcomes, these were narratively reviewed. For continuous outcomes, units were standardised where appropriate (see Supplementary Table 1 for full information), and a random-effects meta-analysis was subsequently conducted with the restricted maximum likelihood method, with heterogeneity quantified using *τ* ^2^. Prediction intervals were also created. Publication bias was assessed with funnel plot asymmetry unless there were fewer than ten component studies, in which case publication bias was not assessed.^23^ Adverse events were pooled using the terminology reported in the original trial publications, with no reclassification of adverse event definitions undertaken. Serious adverse events were defined according to the criteria used in each individual trial. Adverse events were also meta-analysed if there were three or more studies reporting the same adverse events. For adverse events, events and non-events were converted into risk ratios, with log- transformed risk ratios meta-analysed using the same methods as for continuous outcomes and back-transformed into risk ratios for interpretation.

### Risk-of-bias assessment

Risk of bias for each study was determined with the Cochrane Risk of Bias 2 (RoB2) tool,^24^ by two independent researchers (M.T. and K.V.). Although a senior researcher (D.S.) was available to arbitrate any disputes, this was not required as agreement between the two independent researchers was 100%. Supplementary material for each component study was also reviewed.

### Certainty assessment

The certainty of meta-analysed outcomes was assessed with the Grading of Recommendations Assessment, Development and Evaluation (GRADE) approach,^25^ rating outcomes in terms of risk of bias, inconsistency, indirectness, imprecision and publication bias.

## Results

**Fig. 1:**
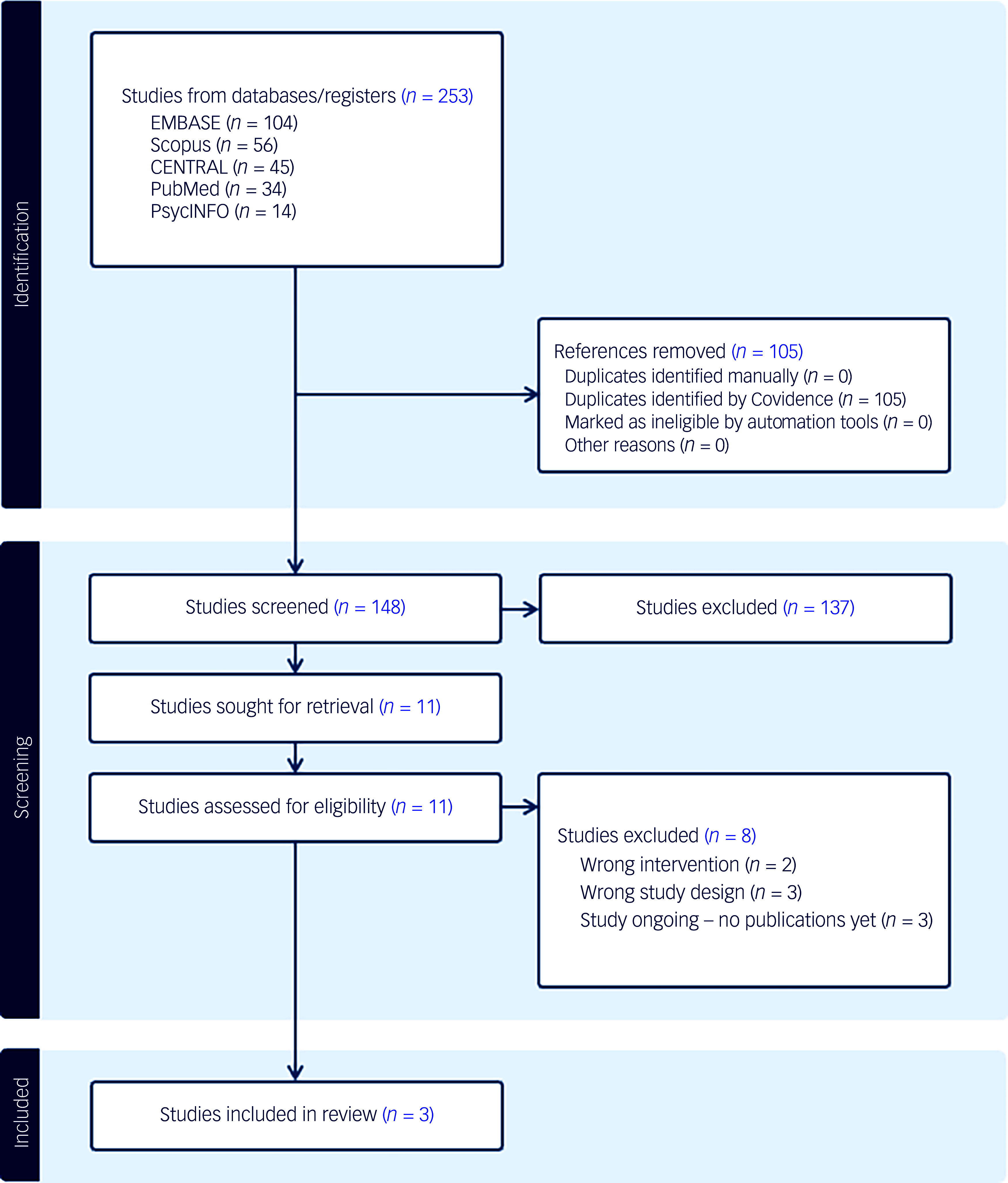
Preferred Reporting of Items for Systematic Reviews and Meta-Analyses (PRISMA) flowchart.

From an initial pool of 253 studies, 148 were screened at the title and abstract level, after which 11 studies were retrieved for full-text review. Following full-text review, three studies satisfied the inclusion criteria and were included in the final review.^21,26,27^ The PRISMA flowchart can be found in Fig. 1 and full reasons for full text exclusion can be found in Supplementary Table 2. Full descriptive information of each component study can be found in Table 1. Two studies were conducted in Denmark,^27,28^ with the third conducted in Australia,^21^ with two studies conducted in community dwelling samples (one study did not specify the setting). All three studies examined semaglutide (total *n* = 258), with two studies examining 1.0 mg dosages and one study examining 2.0 mg. No studies examining tirzepatide were found. Trial durations ranged from 26 to 36 weeks. Regarding the risk of bias of component studies, every study was deemed to have low risk of bias (see Supplementary Table 3 for full information).

**Table 1:** Description of included studies

Authors	Country	Trial registration	Antipsychotic medications	Drug and dosage	Trial duration (weeks)	Inclusion criteria	Total number (treatment/control)	Mean baseline age (s.d.), years, treatment/control	Per cent male, treatment/control
Ganeshalingam et al, 2025^27^	Denmark	NCT05193578	Quetiapine; olanzapine; risperidone; ziprasidone; paliperidone; aripiprazole; clozapine	Semaglutide, 1 mg	30	Aged 18−60 years; diagnosed with schizophrenia, schizotypal disorder or schizoaffective disorder according to the ICD-10; receiving SGA treatment; receiving SGA treatment for at least 6 months without initiation of new antipsychotic medications; participants were required to have HbA1c between 5.7 and 6.4% and a BMI of ≥27 kg/m^2^	154 (77/77)	39 (10.9)/37.6 (10.6)	55/31
Sass et al, 2025^26^	Denmark	NCT04892199	Clozapine; olanzapine	Semaglutide, 1 mg	26	Aged 18−65 years with a schizophrenia spectrum disorder confirmed by the ICD-10 or the DSM-V; initiated treatment with clozapine or olanzapine within the past 60 months; exhibited dysglycaemia at baseline	73 (36/37)	35 (12)/35(13)	36/32
Siskind et al, 2025^21^	Australia	ACTRN12621001539820	Clozapine	Semaglutide, 2 mg	36	18–64 years (inclusive); DSM-IV criteria for schizophrenia or schizoaffective disorder or bipolar affective disorder (however, no one with bipolar affective disorder was recruited); BMI of at least 26 kg/m^2^ at baseline; had received oral clozapine for at least 18 weeks; had less than 5% body weight increase or loss in the previous 3 months	31 (15/16)	39.47 (10.02)/38.31 (8.87)	80/56

SGA, second-generation antipsychotic; HbA1c, haemoglobin A1c; BMI, body mass index.

### Continuous outcomes

#### Meta-analysis

Nine continuous outcomes were collected across all three component studies and included in the meta-analysis. Three outcomes yielded significant results with a high degree of certainty: body mass index (pooled treatment effect: −3.58 kg/m^2^; 95% CI −4.86 to −2.3 kg/m^2^; *p* ≤ 0.001), body weight (pooled treatment effect: −11.32 kg; 95% CI −15.35 to −7.29 kg; *p* ≤ 0.001) and haemoglobin A1c (pooled treatment effect: −0.37%; 95% CI −0.51 to −0.22%; *p* ≤ 0.001). Fasting glucose also yielded a significant treatment effect (−0.54 mmol/L; 95% CI −0.94 to −0.13 mmol/L; *p* = 0.01) at moderate certainty. All other outcomes, outlined in Table 2, were not statically significant, with either low or very low levels of credibility. Full descriptions on how credibility was graded is shown in Supplementary Table 4.

**Table 2:** Meta-analysis results of continuous outcomes

Outcome	Meta-analysis		Heterogeneity		Certainty
	Treatment effect (95% CI)	*p*-value	*τ* ^2^	Prediction interval	
Body mass index (kg/m^2^)	−3.58 (−4.86 to −2.3)	<0.001	1.00	−5.93 to −1.24	High
Body weight (kg)	−11.32 (−15.35 to −7.29)	<0.001	10.15	−18.75 to −3.89	High
Diastolic blood pressure (mmHg)	−0.39 (−2.83 to 2.06)	0.756	0.00	−2.83 to 2.06	Low
Fasting glucose (mmol/L)	−0.54 (−0.94 to −0.13)	0.010	0.09	−1.26 to 0.19	Moderate
Fasting insulin (mU/L)	−3.03 (−7.98 to 1.93)	0.231	1.50	−8.53 to 2.48	Very low
Fasting triglycerides (mmol/L)	−0.14 (−0.41 to 0.13)	0.305	0.00	−0.41 to 0.13	Very low
Haemoglobin A1c (%)	−0.37 (−0.51 to −0.22)	<0.001	0.01	−0.62 to −0.11	High
High-density lipoprotein (mmol/L)	0.07 (−0.1 to 0.24)	0.434	0.02	−0.24 to 0.38	Very low
Systolic blood pressure (mmHg)	−2.34 (−5.53 to 0.84)	0.149	0.00	−5.53 to 0.84	Low

#### Narrative synthesis

Across outcomes not included in the meta-analysis, the most consistent effects were observed for anthropometric and body composition measures. For example, significant and consistent reductions were reported across the two studies that measured body fat percentage and waist circumference, which reported consistent decreases in visceral fat; however, this was not significant in the Sass et al trial.^26^ Furthermore, Siskind et al^21^ reported significant decreases in fat mass, lean mass and body fat percentage, and Ganeshalingam et al^27^ reported significant decreases in hip circumference. In contrast, there were no significant changes in waist/hip ratio and bone mineral content in any of the three trials.

For cardiometabolic and glycaemic outcomes, effects were mostly inconsistent. Two studies reporting on insulin resistance yielded consistent decreases, but neither reached statistical significance. C-peptide demonstrated opposing, non-significant effects across studies, and no clear signal was observed for the Framingham Cardiovascular Index. Similarly, lipid and vascular markers showed no consistent pattern, with non-significant and conflicting effects for low-density lipoprotein and total cholesterol, but consistent reductions in triglycerides and heart rate across studies, albeit without statistical significance. Liver and renal biomarkers were largely unchanged, except for a significant reduction in alkaline phosphatase and significant increase in creatinine, reported by Sass et al.^26^

Across psychiatric symptom severity, cognition and functioning, no consistent evidence of clinical worsening was observed. Psychosis symptoms, measured using the Positive and Negative Syndrome Scale total and subscale scores; overall symptom severity, measured using the Clinical Global Impression − Severity scale; and most areas of cognitive performance, measured with Brief Assessment of Cognition in Schizophrenia, showed non-significant and/or conflicting results across studies. Measures related to substance use and quality of life were similarly mixed, although a significant reduction in nicotine dependence was reported by Sass et al.^26^ Physical activity and sedentary behaviour outcomes were highly variable and imprecise. Finally, clozapine and norclozapine concentrations showed no significant or consistent changes as reported by Siskind et al.^21^ Full information on outcomes not included in the meta-analysis can be found in Table 3 and Supplementary Table 5.

**Table 3:** Clinical, cognitive, and functional outcomes not included in the meta-analysis

Outcome domain	Outcome	Study	Direction versus placebo	Significant
Symptom severity	PANSS total	Siskind et al^21^	No change	No
	PANSS positive	Siskind et al^21^	Decrease	No
	PANSS negative	Siskind et al^21^	Decrease	No
	PANSS general psychopathology	Siskind et al^21^	Increase	No
	PANSS-6	Sass et al^26^	Increase	No
	CGI-S	Sass et al^26^	Increase	No
Cognition	BACS verbal memory learning	Siskind et al^21^	Increase	Yes
	BACS symbol coding	Siskind et al^21^	Decrease	No
	BACS animals	Siskind et al^21^	Decrease	No
	BACS digital sequencing	Siskind et al^21^	Increase	No
	Trail Making Test A (time/errors)	Siskind et al^21^	Mixed	No
	Trail Making Test B (time/errors)	Siskind et al^21^	Mixed	No
Functioning	GAPD	Sass et al^26^	Decrease	No
Quality of life	SQLS psychosocial	Sass et al^26^	Decrease	No
	SQLS motivation	Sass et al^26^	Decrease	No
	SQLS adverse effects	Sass et al^26^	Decrease	No
Substance use	AUDIT	Siskind et al^21^	Decrease	No
	AUDIT	Sass et al^26^	Increase	No
	FTND (nicotine dependence)	Sass et al^26^	Decrease	Yes

PANSS, Positive and Negative Syndrome Scale; CGI-S, Clinical Global Impression–Severity; BACS, Brief Assessment of Cognition in Schizophrenia; GAPD, Global Assessment of Psychosocial Disability; SQLS, Schizophrenia Quality of Life Scale; AUDIT, Alcohol Use Disorders Identification Test; FTND, Fagerström Test for Nicotine Dependence.

### Adverse events

#### Meta-analysis

**Fig. 2:**
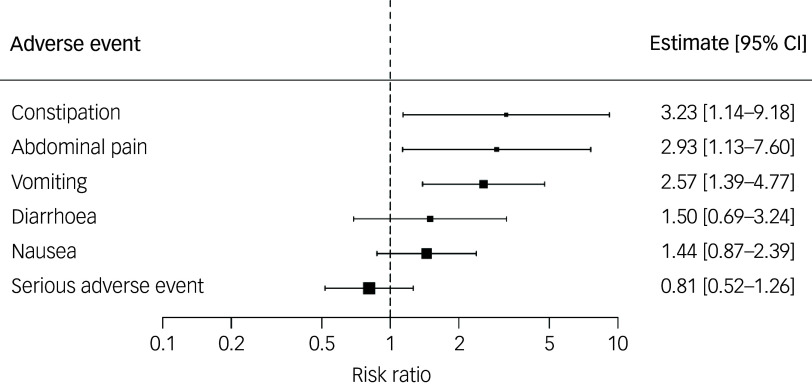
Forest plot showing risks of adverse events in semaglutide versus placebo groups.

Six types of adverse events were reported in enough studies to be included in the meta-analysis: any serious adverse events, abdominal pain, vomiting, diarrhoea, nausea and constipation (one study did not report ‘any adverse events’ and therefore was not included in the meta-analysis). The risks of abdominal pain (risk ratio 2.93; 95% CI 1.13−7.60; *p* = 0.03) and vomiting (risk ratio 2.57; 95% CI 1.39−4.77; *p* ≤ 0.01) were significantly higher in the semaglutide group, with a high degree of certainty. Furthermore, the risk of constipation (risk ratio 3.23; 95% CI 1.14−9.18; *p* = 0.03) was significantly higher in the semaglutide group, with a moderate degree of certainty. All other adverse event outcomes did not significantly differ between groups. Full information can be found in Table 4 and Fig. 2, and details on how certainty was graded are in Supplementary Table 6.

**Table 4:** Meta-analysis results of adverse events

Outcome	Meta-analysis		Heterogeneity		Certainty
	Risk ratio (95% CI)	*p*-value	*τ* ^2^	Prediction interval	
Abdominal pain	2.93 (1.13–7.6)	0.027	0	1.13–7.60	High
Constipation	3.23 (1.14–9.18)	0.028	0.38	0.65–16.03	Moderate
Diarrhoea	1.5 (0.69–3.24)	0.308	0.14	0.52–4.34	Low
Nausea	1.44 (0.87–2.39)	0.152	0.08	0.69–3.02	Low
Serious adverse event	0.81 (0.52–1.26)	0.345	0	0.52–1.26	Low
Vomiting	2.57 (1.39–4.77)	0.003	0	1.39–4.77	High

#### Narrative synthesis

No consistent or replicated adverse events were observed across studies in terms of the non-meta-analysed adverse events. Most adverse events were reported by single studies, with study-level risk ratios being highly imprecise because of low event counts. Rates of any adverse event and adverse events leading to discontinuation were broadly comparable between semaglutide and control groups, with risk ratios close to unity where reported, suggesting no clear excess overall treatment-related harm.

Where individual adverse events were reported in more than one study, direction of effect was frequently inconsistent, particularly for gastrointestinal and neurological symptoms such as dizziness, fatigue, headache and flatulence. Gastrointestinal events were common across studies, but showed variable effect sizes and limited reproducibility. Psychiatric adverse events, including psychiatric admissions, self-harm and suicidal behaviour, were infrequently reported and did not demonstrate a consistent pattern of increased risk. Importantly, no consistent changes in serious adverse events, infections or mortality-related outcomes were observed. Full adverse event data can be found in Supplementary Table 7.

## Discussion

This systematic review and meta-analysis synthesised the published RCTs evaluating semaglutide and/or tirzepatide in people with SSDs. None of the identified trials in this population examined tirzepatide. In all three included trials, semaglutide yielded clinically meaningful improvements in weight and glycaemic outcomes, with high certainty evidence for reductions in body mass index, body weight and haemoglobin A1c, and moderate certainty for fasting glucose, compared with placebo. Across outcomes that were not meta-analysed, semaglutide was also associated with broadly consistent improvements across adiposity-related measures, including body fat percentage, waist circumference and visceral fat. These reductions in weight and glycaemic outcomes are notable, considering the substantial cardiometabolic burden in people with SSDs^3^ and the limited comparative efficacy of existing adjunctive strategies. For example, weight loss from placebo-controlled metformin trials is 3 kg compared with 11 kg with semaglutide in the current review.^29^ These findings are broadly consistent with treatment effects reported for obesity management with semaglutide within the general population, reinforcing the potential of this drug class as an effective pharmacological strategy for addressing antipsychotic-associated metabolic dysfunction.^18,30^ Notably, the 11% reduction in body weight observed in this review provides preliminary evidence that similar weight-loss effects may be achievable in people with SSDs, despite the use of a lower semaglutide doses (1.0−2.0 mg compared with 2.4 mg in general population trials) and shorter trial durations (68−104 weeks in general population studies versus 26−36 weeks across the three component RCTs).^18,30^ Importantly, there was also no consistent evidence of psychiatric deterioration or reductions in function, reducing concern about major pharmacokinetic interactions in people with SSDs. From a clinical perspective, these findings support semaglutide as a promising adjunctive metabolic intervention for people with SSDs, particularly where obesity and indices of metabolic syndrome threaten long-term health and antipsychotic switching is infeasible.

Several mechanistic features support the potential utility of GLP-1 RA-based therapies in people with SSDs. Importantly, their metabolic actions occur independently of the dopaminergic and serotonergic pathways targeted by antipsychotic medications, reducing the likelihood of direct pharmacological interaction, as evidenced in this review. Furthermore, preclinical studies suggest that antipsychotic exposure (particularly clozapine and olanzapine) may suppress endogenous GLP-1 signalling, with downstream effects on hepatic glucose production, glucagon secretion and appetite regulation.^31^ Collectively, these mechanisms provide a biologically plausible rationale for GLP-1 RA therapy as an adjunct treatment to counteract antipsychotic-associated metabolic dysfunction.

Regarding safety, meta-analysed adverse events indicated increased risks of gastrointestinal events, including abdominal pain, vomiting and constipation, with moderate-to-high certainty. In contrast, there was no evidence of an increased risk of serious adverse events. This adverse event profile is consistent with the tolerability profile of semaglutide as seen in large studies in the general population.^18,30^ The consistent findings of gastrointestinal adverse events, however, have practical implications for prescribing. In SSD populations, adverse events that affect hydration, appetite and treatment adherence may be particularly consequential,^32,33^ and implementation should therefore include pragmatic safeguards: careful dose escalation, anticipatory management of constipation and nausea/vomiting, monitoring for dehydration and reduced oral intake, and proactive follow-up in the early weeks of treatment. This is of particular importance to people receiving clozapine, which has an enhanced adverse event profile compared with some other antipsychotic medications.^34^ The timing of adverse events relative to dose escalation is also an important factor to consider, and was not reported in a any of the component studies. Future research should aim to include this level of adverse event information because of its clinical relevance. Furthermore, considering the high baseline physical comorbidity in people with SSDs,^35^ clinicians should also consider routine metabolic monitoring and a low threshold for review where symptoms suggest intolerance or emerging complications.

The results of this review should be interpreted considering some limitations. First, only three trials were available, limiting precision for many outcomes, reducing the ability to explore effect modifiers and limiting the confident interpretation of heterogeneity and prediction intervals. Second, antipsychotic exposure may influence interpretation of the findings. Clozapine and olanzapine, for example, are associated with particularly high cardiometabolic risk,^5^ with one included trial exclusively recruiting individuals treated with clozapine and another with clozapine and/or olanzapine. Although this increases the clinical relevance of the findings, heterogeneity in antipsychotic treatment across trials may contribute to differences in baseline metabolic risk and treatment response. Third, follow-up ranged from 26 to 36 weeks, which is only sufficient to demonstrate short-term outcomes, not to characterise longer-term durability, maintenance strategies or outcomes such as incident diabetes, cardiovascular events or mortality. Fourth, adverse event reporting varied across trials, with many adverse event types having low cell counts or inconsistently defined, thereby leaving uncertainty around less common, but clinically relevant outcomes. Fifth, the evidence base relates specifically to semaglutide at 1.0–2.0 mg, with no trials of tirzepatide being identified, representing a major gap considering tirzepatide’s comparative efficacy versus semaglutide in weight loss in non-SSD populations.^20^ Finally, although all included studies were judged to be low risk of bias, the total sample was low, and therefore results should be interpreted with caution.

Future research should prioritise larger, longer trials with harmonised outcome sets and robust safety reporting, including stratification by antipsychotic class (particularly clozapine/olanzapine), baseline metabolic status and key potential moderators of adherence. Trials should also examine implementation questions that matter clinically: optimal titration and tolerability protocols in SSDs, strategies to support adherence in the presence of gastrointestinal-related adverse events, comparative effectiveness against metformin and/or combined approaches, and whether improvements in adiposity translate into downstream cardiometabolic and functional outcomes. Considering that GLP-1 RAs markedly suppress appetite, leading to substantial reductions in overall food intake, it would be prudent to evaluate the quality of dietary intake and potential risk of micronutrient deficiencies during administration. Trials that explore co-commencement of GLP-1 RAs at time of antipsychotic initiation to ameliorate weight gain are needed. Furthermore, evidence on the efficacy and safety of tirzepatide is urgently needed in this population as no studies were found. Finally, aiming for an effective long-term management of antipsychotic-induced weight gain, studies should also explore combining pharmacological treatment with GLP-1 RAs with behavioural approaches targeting key lifestyle behaviours, including nutrition and physical activity, which may help to maximise the effects of both treatment approaches and improve cardiometabolic health in the long term.^36^

In conclusion, the current RCT evidence supports semaglutide as an effective adjunctive therapy to improve weight and glycaemic outcomes in people with SSDs, with adverse events characterised by gastrointestinal effects and no signal for serious adverse events in pooled analyses. Although these findings are clinically promising, larger trials of longer duration are required to define durability, identify who benefits most and more comprehensively evaluate long-term safety in this high-risk population.

## Supporting information

10.1192/bjo.2026.12001.sm001 Trott et al. supplementary material Trott et al. supplementary material

## Data Availability

Data availability is not applicable to this article as no new data were created or analysed in this study. All analytical code is available upon reasonable request.

## Supplementary material

The supplementary material is available online at https://doi.org/10.1192/bjo.2026.12001

## References

[1] Vigo D , Thornicroft G , Atun R. Estimating the true global burden of mental illness. Lancet Psychiatry 2016; 3: 171–8. 26851330 10.1016/S2215-0366(15)00505-2

[2] GBD 2019 Mental Disorders Collaborators. Global, regional, and national burden of 12 mental disorders in 204 countries and territories, 1990–2019: a systematic analysis for the Global Burden of Disease Study 2019. Lancet Psychiatry 2022; 9: 137–50. 35026139 10.1016/S2215-0366(21)00395-3 PMC8776563

[3] Correll CU , Solmi M , Veronese N , Bortolato B , Rosson S , Santonastaso P , et al. Prevalence, incidence and mortality from cardiovascular disease in patients with pooled and specific severe mental illness: a large-scale meta-analysis of 3,211,768 patients and 113,383,368 controls. World Psychiatry 2017; 16: 163–80. 28498599 10.1002/wps.20420 PMC5428179

[4] Tek C , Kucukgoncu S , Guloksuz S , Woods SW , Srihari VH , Annamalai A. Antipsychotic-induced weight gain in first-episode psychosis patients: a meta-analysis of differential effects of antipsychotic medications. Focus 2016; 14: 370–7. 31997958 10.1176/appi.focus.140308 PMC6988756

[5] Burschinski A , Schneider‐Thoma J , Chiocchia V , Schestag K , Wang D , Siafis S , et al. Metabolic side effects in persons with schizophrenia during mid-to long-term treatment with antipsychotics: a network meta-analysis of randomized controlled trials. World Psychiatry 2023; 22: 116–28. 36640396 10.1002/wps.21036 PMC9840505

[6] Siskind D , Gallagher E , Winckel K , Hollingworth S , Kisely S , Firth J , et al. Does switching antipsychotics ameliorate weight gain in patients with severe mental illness? A systematic review and meta-analysis. Schizophrenia Bull 2021; 47: 948–58. 10.1093/schbul/sbaa191 PMC8266669 33547471

[7] Townsend M , Pareja K , Buchanan-Hughes A , Worthington E , Pritchett D , Brubaker M , et al. Antipsychotic-related stigma and the impact on treatment choices: a systematic review and framework synthesis. Patient Prefer Adher 2022; 16: 373–401. 10.2147/PPA.S343211 PMC8859276 35210756

[8] De R , Smith ECC , Navagnanavel J , Au E , Maksyutynska K , Papoulias M , et al. The impact of weight gain on antipsychotic nonadherence or discontinuation: a systematic review and meta-analysis. Acta Psychiat Scand 2025; 151: 109–26. 39285800 10.1111/acps.13758 PMC11695092

[9] Masand PS , Gupta S. Quality of life issues associated with antipsychotic-induced weight gain. Expert Rev Pharm Out 2003; 3: 651–9. 10.1586/14737167.3.5.651 19807398

[10] Stevens H , Smith J , Bussey L , Innerd A , McGeechan G , Fishburn S , et al. Weight management interventions for adults living with overweight or obesity and severe mental illness: a systematic review and meta-analysis. Br J Nutr 2023; 130: 536–52. 36325987 10.1017/S0007114522003403 PMC10331435

[11] Lee C , Piernas C , Stewart C , Michalopoulou M , Hajzadeh A , Edwards R , et al. Identifying effective characteristics of behavioral weight management interventions for people with serious mental illness: a systematic review with a qualitative comparative analysis. Obes Rev 2022; 23: e13355. 34672069 10.1111/obr.13355 PMC8952200

[12] Yu O , Lu M , Lai TKY , Hahn M , Agarwal SM , O’Donoghue B , et al. Metformin co-commencement at time of antipsychotic initiation for attenuation of weight gain: a systematic review and meta-analysis. Ther Adv Psychopharmacol 2024; 14: 20451253241255476. 38827016 10.1177/20451253241255476 PMC11141220

[13] Vancampfort D , Firth J , Correll CU , Solmi M , Siskind D , De Hert M , et al. The impact of pharmacological and non-pharmacological interventions to improve physical health outcomes in people with schizophrenia: a meta-review of meta-analyses of randomized controlled trials. Focus 2021; 19: 116–28. 34483776 10.1176/appi.focus.19103 PMC8412153

[14] Nauck MA , Heimesaat MM , Behle K , Holst JJ , Nauck MS , Ritzel R , et al. Effects of glucagon-like peptide 1 on counterregulatory hormone responses, cognitive functions, and insulin secretion during hyperinsulinemic, stepped hypoglycemic clamp experiments in healthy volunteers. J Clin Endocrinol Metab 2002; 87: 1239–46. 11889194 10.1210/jcem.87.3.8355

[15] Doyle M , Egan J. Mechanisms of action of GLP-1 in the pancreas. Pharmacol Ther 2007; 113: 546–93. 17306374 10.1016/j.pharmthera.2006.11.007 PMC1934514

[16] Vosoughi K , Atieh J , Khanna L , Khoshbin K , Prokop LJ , Davitkov P , et al. Association of glucagon-like peptide 1 analogs and agonists administered for obesity with weight loss and adverse events: a systematic review and network meta-analysis. EClinicalMedicine 2021; 42: 101213. 34877513 10.1016/j.eclinm.2021.101213 PMC8633575

[17] Siskind D , Hahn M , Correll CU , Fink‐Jensen A , Russell AW , Bak N , et al. Glucagon-like peptide-1 receptor agonists for antipsychotic-associated cardio-metabolic risk factors: a systematic review and individual participant data meta-analysis. Diabetes Obes Metab 2019; 21: 293–302. 30187620 10.1111/dom.13522

[18] Wilding JPH , Batterham RL , Calanna S , Davies M , Van Gaal LF , Lingvay I , et al. Once-weekly semaglutide in adults with overweight or obesity. New Engl J Med 2021; 384: 989–1002. 33567185 10.1056/NEJMoa2032183

[19] Jastreboff AM , Aronne LJ , Ahmad NN , Wharton S , Connery L , Alves B , et al. Tirzepatide once weekly for the treatment of obesity. N Engl J Med 2022; 387: 205–16. 35658024 10.1056/NEJMoa2206038

[20] Aronne LJ , Horn DB , le Roux CW , Ho W , Falcon BL , Gomez Valderas E , et al. Tirzepatide as compared with semaglutide for the treatment of obesity. N Engl J Med 2025; 393: 26–36. 40353578 10.1056/NEJMoa2416394

[21] Siskind D , Baker A , Arnautovska U , Warren N , Russell A , DeMonte V , et al. Efficacy and safety of semaglutide versus placebo for people with schizophrenia on clozapine with obesity (COaST): a phase 2, multi-centre, participant and investigator- blinded, randomised controlled trial in Australia. Lancet Psychiatry 2025; 12: 493–503. 40506208 10.1016/S2215-0366(25)00129-4

[22] Page MJ , Moher D , Bossuyt PM , Boutron I , Hoffmann TC , Mulrow CD , et al. PRISMA 2020 explanation and elaboration: updated guidance and exemplars for reporting systematic reviews. BMJ 2020; 2021: 372. 10.1136/bmj.n160 PMC8005925 33781993

[23] Higgins JP , Green C. Handbook for Systematic Reviews of Interventions. Cochrane Collaboration, 2008.

[24] Sterne JA C , Savović J , Page MJ , Elbers RG , Blencowe NS , Boutron I , et al. RoB 2: a revised tool for assessing risk of bias in randomised trials. BMJ 2019; 366: l4898. 31462531 10.1136/bmj.l4898

[25] Zhang Y , Akl EA , Schünemann HJ . Using systematic reviews in guideline development: the GRADE approach. Res Synth Methods [Epub ahead of print] 14 Jul 2018. Available from: 10.1002/jrsm.1313. 30006970

[26] Sass MR , Klausen MK , Schwarz CR , Rasmussen L , Giver MEB , Hviid M , et al. Semaglutide and early-stage metabolic abnormalities in individuals with schizophrenia spectrum disorders: a randomized clinical trial. JAMA Psychiatry 2025; 83: 128–38. 10.1001/jamapsychiatry.2025.3639 PMC12676471 41335431

[27] Ganeshalingam AA , Uhrenholt N , Arnfred S , Gæde P , Düring S , Stenager EN , et al. Semaglutide treatment of antipsychotic-treated patients with schizophrenia, prediabetes, and obesity: the HISTORI randomized clinical trial. JAMA Psychiatry 2025; 82: 1065–74. 40900607 10.1001/jamapsychiatry.2025.2332 PMC12409653

[28] Fink-Jensen A , Wörtwein G , Klausen MK , Holst JJ , Hartmann B , Thomsen M , et al. Effect of the glucagon-like peptide-1 (GLP-1) receptor agonist semaglutide on alcohol consumption in alcohol-preferring male vervet monkeys. Psychopharmacology (Berl) 2025; 242: 63–70. 38884652 10.1007/s00213-024-06637-2 PMC11742737

[29] Siskind DJ , Leung J , Russell AW , Wysoczanski D , Kisely S , Holscher C. Metformin for clozapine associated obesity: a systematic review and meta-analysis. PLOS One 2016; 11: e0156208. 27304831 10.1371/journal.pone.0156208 PMC4909277

[30] Lincoff AM , Brown-Frandsen K , Colhoun HM , Deanfield J , Emerson SS , Esbjerg S , et al. Semaglutide and cardiovascular outcomes in obesity without diabetes. N Engl J Med 2023; 389: 2221–32. 37952131 10.1056/NEJMoa2307563

[31] Smith GC , Vickers MH , Cognard E , Shepherd PR. Clozapine and quetiapine acutely reduce glucagon-like peptide-1 production and increase glucagon release in obese rats: implications for glucose metabolism and food choice behaviour. Schizophr Res 2009; 115: 30–40. 19679451 10.1016/j.schres.2009.07.011

[32] Schneider-Thoma J , Efthimiou O , Bighelli I , Dörries C , Huhn M , Krause M , et al. Second-generation antipsychotic drugs and short-term somatic serious adverse events: a systematic review and meta-analysis. Lancet Psychiatry 2019; 6: 753–65. 31320283 10.1016/S2215-0366(19)30223-8

[33] De Hert M , Detraux J , van Winkel R , Yu W , Correll CU. Metabolic and cardiovascular adverse effects associated with antipsychotic drugs. Nat Rev Endocrinol 2012; 8: 114–26. 10.1038/nrendo.2011.156 22009159

[34] Tanzer T , Pham B , Warren N , Barras M , Kisely S , Siskind D. Overcoming clozapine’s adverse events: a narrative review of systematic reviews and meta-analyses. Expert Opin Drug Saf 2024; 23: 811–31. 38814794 10.1080/14740338.2024.2362796

[35] Trott M , Plever S , Anzolin M , McCarthy I , Siskind D. Rates of metabolic syndrome in Queensland adult community mental health consumers with schizophrenia and related disorders: a brief report. Australas Psychiatry 2025; 33: 731–5. 40616255 10.1177/10398562251358760 PMC12314204

[36] Arnautovska U , Milton A , Trott M , Soole R , Siskind D. The role of human involvement and support in digital mental health interventions for people with schizophrenia spectrum disorders: a critical review. Curr Opin Psychiatry 2024; 37: 356–62. 38994811 10.1097/YCO.0000000000000957

