# Supplementary material for: The safety and efficacy of semaglutide in people with schizophrenia spectrum disorders: systematic review and meta-analysis of randomised controlled trials
Source: BJPsych Open. 2026 May 25;12(3):e147. doi: 10.1192/bjo.2026.12001 (PMC13202607; doi:10.1192/bjo.2026.12001)
Supplement: Trott et al. supplementary material [file S2056472426120018sup001.docx]

**PRISMA checklist**

| **Section and Topic** | **Item #** | **Checklist item** | **Location where item is reported** |
| --- | --- | --- | --- |
| **TITLE** | | |  |
| Title | 1 | Identify the report as a systematic review. | Title page (page 1), abstract (page 2), and page 4. |
| **ABSTRACT** | | |  |
| Abstract | 2 | See the PRISMA 2020 for Abstracts checklist. | Page 2 |
| **INTRODUCTION** | | |  |
| Rationale | 3 | Describe the rationale for the review in the context of existing knowledge. | Pages 3-4 |
| Objectives | 4 | Provide an explicit statement of the objective(s) or question(s) the review addresses. | Page 4 |
| **METHODS** | | |  |
| Eligibility criteria | 5 | Specify the inclusion and exclusion criteria for the review and how studies were grouped for the syntheses. | Page 5 |
| Information sources | 6 | Specify all databases, registers, websites, organisations, reference lists and other sources searched or consulted to identify studies. Specify the date when each source was last searched or consulted. | Page 4 |
| Search strategy | 7 | Present the full search strategies for all databases, registers and websites, including any filters and limits used. | Supplementary Materials |
| Selection process | 8 | Specify the methods used to decide whether a study met the inclusion criteria of the review, including how many reviewers screened each record and each report retrieved, whether they worked independently, and if applicable, details of automation tools used in the process. | Page 4 |
| Data collection process | 9 | Specify the methods used to collect data from reports, including how many reviewers collected data from each report, whether they worked independently, any processes for obtaining or confirming data from study investigators, and if applicable, details of automation tools used in the process. | Page 4 |
| Data items | 10a | List and define all outcomes for which data were sought. Specify whether all results that were compatible with each outcome domain in each study were sought (e.g. for all measures, time points, analyses), and if not, the methods used to decide which results to collect. | Page 4 |
|  | 10b | List and define all other variables for which data were sought (e.g. participant and intervention characteristics, funding sources). Describe any assumptions made about any missing or unclear information. | Page 4 |
| Study risk of bias assessment | 11 | Specify the methods used to assess risk of bias in the included studies, including details of the tool(s) used, how many reviewers assessed each study and whether they worked independently, and if applicable, details of automation tools used in the process. | Page 5 |
| Effect measures | 12 | Specify for each outcome the effect measure(s) (e.g. risk ratio, mean difference) used in the synthesis or presentation of results. | Page 5 |
| Synthesis methods | 13a | Describe the processes used to decide which studies were eligible for each synthesis (e.g. tabulating the study intervention characteristics and comparing against the planned groups for each synthesis (item #5)). | Page 5 |
|  | 13b | Describe any methods required to prepare the data for presentation or synthesis, such as handling of missing summary statistics, or data conversions. | Pages 4 and 5 |
|  | 13c | Describe any methods used to tabulate or visually display results of individual studies and syntheses. | Page 5 |
|  | 13d | Describe any methods used to synthesize results and provide a rationale for the choice(s). If meta-analysis was performed, describe the model(s), method(s) to identify the presence and extent of statistical heterogeneity, and software package(s) used. | Page 5 |
|  | 13e | Describe any methods used to explore possible causes of heterogeneity among study results (e.g. subgroup analysis, meta-regression). | Page 5 |
|  | 13f | Describe any sensitivity analyses conducted to assess robustness of the synthesized results. | Page 5 |
| Reporting bias assessment | 14 | Describe any methods used to assess risk of bias due to missing results in a synthesis (arising from reporting biases). | Page 5 |
| Certainty assessment | 15 | Describe any methods used to assess certainty (or confidence) in the body of evidence for an outcome. | Page 5 |
| **RESULTS** | | |  |
| Study selection | 16a | Describe the results of the search and selection process, from the number of records identified in the search to the number of studies included in the review, ideally using a flow diagram. | Page 6 |
|  | 16b | Cite studies that might appear to meet the inclusion criteria, but which were excluded, and explain why they were excluded. | Page 6 and Supplementary Materials |
| Study characteristics | 17 | Cite each included study and present its characteristics. | Page 6 |
| Risk of bias in studies | 18 | Present assessments of risk of bias for each included study. | Page 6 |
| Results of individual studies | 19 | For all outcomes, present, for each study: (a) summary statistics for each group (where appropriate) and (b) an effect estimate and its precision (e.g. confidence/credible interval), ideally using structured tables or plots. | Pages 6 and 7 |
| Results of syntheses | 20a | For each synthesis, briefly summarise the characteristics and risk of bias among contributing studies. | Pages 6 and 7 |
|  | 20b | Present results of all statistical syntheses conducted. If meta-analysis was done, present for each the summary estimate and its precision (e.g. confidence/credible interval) and measures of statistical heterogeneity. If comparing groups, describe the direction of the effect. | Pages 6 and 7 |
|  | 20c | Present results of all investigations of possible causes of heterogeneity among study results. | Pages 6 and 7 |
|  | 20d | Present results of all sensitivity analyses conducted to assess the robustness of the synthesized results. | Pages 6 and 7 |
| Reporting biases | 21 | Present assessments of risk of bias due to missing results (arising from reporting biases) for each synthesis assessed. | Pages 6 and 7 |
| Certainty of evidence | 22 | Present assessments of certainty (or confidence) in the body of evidence for each outcome assessed. | Pages 6 and 7 |
| **DISCUSSION** | | |  |
| Discussion | 23a | Provide a general interpretation of the results in the context of other evidence. | Pages 9-11 |
|  | 23b | Discuss any limitations of the evidence included in the review. | Pages 9-11 |
|  | 23c | Discuss any limitations of the review processes used. | Pages 9-11 |
|  | 23d | Discuss implications of the results for practice, policy, and future research. | Pages 9-11 |
| **OTHER INFORMATION** | | |  |
| Registration and protocol | 24a | Provide registration information for the review, including register name and registration number, or state that the review was not registered. | Abstract (page 2) and page 4 |
|  | 24b | Indicate where the review protocol can be accessed, or state that a protocol was not prepared. | Abstract (page 2) and page 4 |
|  | 24c | Describe and explain any amendments to information provided at registration or in the protocol. | Page 4 |
| Support | 25 | Describe sources of financial or non-financial support for the review, and the role of the funders or sponsors in the review. | Title page (page 1) |
| Competing interests | 26 | Declare any competing interests of review authors. | Title page (page 1) |
| Availability of data, code and other materials | 27 | Report which of the following are publicly available and where they can be found: template data collection forms; data extracted from included studies; data used for all analyses; analytic code; any other materials used in the review. | Title page (page 1) |

*From:*  Page MJ, McKenzie JE, Bossuyt PM, Boutron I, Hoffmann TC, Mulrow CD, et al. The PRISMA 2020 statement: an updated guideline for reporting systematic reviews. BMJ 2021;372:n71. doi: 10.1136/bmj.n71. This work is licensed under CC BY 4.0. To view a copy of this license, visit <https://creativecommons.org/licenses/by/4.0/>

**Search criteria**

(semaglutide OR tirzepatide OR "GLP-1 receptor agonist" OR "GLP1 receptor agonist"

OR "glucagon-like peptide-1" OR Ozempic OR Wegovy OR Rybelsus OR Mounjaro)

AND

(schizophrenia OR psychosis OR psychotic OR schizoaffective)

AND

(randomized controlled trial OR randomised controlled trial OR randomized OR randomised OR placebo OR RCT)

**Supplementary Table 1**: Standardisation of units for outcome

| Outcome | Author | Original metric | Converted metric | Conversation equation |
| --- | --- | --- | --- | --- |
| Fasting glucose | Sass et al. | mg/dL | mmol/L | mmol/L = mg/dL/18.02 |
|  | Ganeshalingam et al. | mg/dL | mmol/L |  |
| HDL cholesterol | Sass et al. | mg/dL | mmol/L | mmol/L = mg/dL/38.67 |
|  | Ganeshalingam et al. | mg/dL | mmol/L |  |
| Fasting triglycerides | Sass et al. | mg/dL | mmol/L | mmol/L = mg/dL/88.57 |
|  | Ganeshalingam et al. | mg/dL | mmol/L |  |
| Visceral fat | Sass et al. | g | kg | kg=g/1000 |

HDL=high density lipoprotein

**Supplementary Table 2**: Full list of excluded studies with reasons for exclusion

| Authors | Title | Reason for exclusion |
| --- | --- | --- |
| Bergen et al. (2024) | An interventional, multi-center, randomized, double blinded, placebo controlled study to investigate semaglutide add-on treatment for metabolic control in antipsychotic-using patients (STABIL-NOR ‚Äì study) | Clinical trial protocol. |
| Tomasik et al. (2020) | Leptin Serum Levels are Associated With GLP-1 Receptor Agonist-Mediated Effects on Glucose Metabolism in Clozapine-or Olanzapine-Treated, Prediabetic, Schizophrenia Patients | Study design. |
| Caiji et al (2025) | Semaglutide versus metformin in patients with schizophrenia and metabolic syndrome: a randomized, open-controlled study | Clinical trial protocol. |
| Zhang et al. (2025) | Exploring glucagon-like peptide-1 receptor agonists as potential disease-modifying agent in psychiatric and neurodevelopmental conditions: evidence from a drug target Mendelian randomization | Study design. |
| Jacobsen et al. (2025) | Efficacy and safety of GLP1-ras compared to SGLT2is and DPP-4is in individuals with schizophrenia and diabetes: A Danish nationwide target-trial emulation study | Study design. |
| Hahn et al (2022) | Semaglutide in Comorbid Schizophrenia Spectrum Disorder and Obesity for Metformin Non-responders: a Single-blind Randomized Control Trial | Clinical trial protocol. |
| Whicher et al. (2021) | The use of liraglutide 3.0¬†mg daily in the management of overweight and obesity in people with schizophrenia, schizoaffective disorder and first episode psychosis: Results of a pilot randomized, double-blind, placebo-controlled trial | Wrong intervention. |
| Xiang and Peng (2025) | Impact of Glucagon-like Peptide-1 Receptor Agonists on Mental Illness: Evidence from a Mendelian Randomization Study | Study design. |

**Supplementary Table 3**: Risk of bias results

| Unique ID | 1 | Study ID | Sass et al. | | |
| --- | --- | --- | --- | --- | --- |
| Domain | Signalling question | | | Response | Comments |
| Bias arising from the randomization process | 1.1 Was the allocation sequence random? | | | Y |  |
|  | 1.2 Was the allocation sequence concealed until participants were enrolled and assigned to interventions? | | | Y |  |
|  | 1.3 Did baseline differences between intervention groups suggest a problem with the randomization process? | | | N |  |
|  | Risk of bias judgement | | | Low |  |
| Bias due to deviations from intended interventions | 2.1.Were participants aware of their assigned intervention during the trial? | | | PY | The effect size was so large vs placebo there is the possibility participants would have known |
|  | 2.2.Were carers and people delivering the interventions aware of participants' assigned intervention during the trial? | | | N |  |
|  | 2.3. If Y/PY/NI to 2.1 or 2.2: Were there deviations from the intended intervention that arose because of the experimental context? | | | N |  |
|  | 2.4 If Y/PY to 2.3: Were these deviations likely to have affected the outcome? | | | NA |  |
|  | 2.5. If Y/PY/NI to 2.4: Were these deviations from intended intervention balanced between groups? | | | NA |  |
|  | 2.6 Was an appropriate analysis used to estimate the effect of assignment to intervention? | | | Y |  |
|  | 2.7 If N/PN/NI to 2.6: Was there potential for a substantial impact (on the result) of the failure to analyse participants in the group to which they were randomized? | | | NA |  |
|  | Risk of bias judgement | | | Low |  |
| Bias due to missing outcome data | 3.1 Were data for this outcome available for all, or nearly all, participants randomized? | | | Y |  |
|  | 3.2 If N/PN/NI to 3.1: Is there evidence that result was not biased by missing outcome data? | | | NA |  |
|  | 3.3 If N/PN to 3.2: Could missingness in the outcome depend on its true value? | | | NA |  |
|  | 3.4 If Y/PY/NI to 3.3: Is it likely that missingness in the outcome depended on its true value? | | | NA |  |
|  | Risk of bias judgement | | | Low |  |
| Bias in measurement of the outcome | 4.1 Was the method of measuring the outcome inappropriate? | | | N |  |
|  | 4.2 Could measurement or ascertainment of the outcome have differed between intervention groups? | | | N |  |
|  | 4.3 Were outcome assessors aware of the intervention received by study participants? | | | N |  |
|  | 4.4 If Y/PY/NI to 4.3: Could assessment of the outcome have been influenced by knowledge of intervention received? | | | NA |  |
|  | 4.5 If Y/PY/NI to 4.4: Is it likely that assessment of the outcome was influenced by knowledge of intervention received? | | | NA |  |
|  | Risk of bias judgement | | | Low |  |
| Bias in selection of the reported result | 5.1 Were the data that produced this result analysed in accordance with a pre-specified analysis plan that was finalized before unblinded outcome data were available for analysis? | | | Y |  |
|  | 5.2 ... multiple eligible outcome measurements (e.g. scales, definitions, time points) within the outcome domain? | | | N |  |
|  | 5.3 ... multiple eligible analyses of the data? | | | N |  |
|  | Risk of bias judgement | | | Low |  |
| Overall bias | Risk of bias judgement | | | Low |  |
| Unique ID | 2 | Study ID | Siskind et al. | | |
| Domain | Signalling question | | | Response | Comments |
| Bias arising from the randomization process | 1.1 Was the allocation sequence random? | | | Y |  |
|  | 1.2 Was the allocation sequence concealed until participants were enrolled and assigned to interventions? | | | Y |  |
|  | 1.3 Did baseline differences between intervention groups suggest a problem with the randomization process? | | | N |  |
|  | Risk of bias judgement | | | Low |  |
| Bias due to deviations from intended interventions | 2.1.Were participants aware of their assigned intervention during the trial? | | | PN | The effect size was so large vs placebo there is the possibility participants would have known |
|  | 2.2.Were carers and people delivering the interventions aware of participants' assigned intervention during the trial? | | | Y |  |
|  | 2.3. If Y/PY/NI to 2.1 or 2.2: Were there deviations from the intended intervention that arose because of the experimental context? | | | Y |  |
|  | 2.4 If Y/PY to 2.3: Were these deviations likely to have affected the outcome? | | | N |  |
|  | 2.5. If Y/PY/NI to 2.4: Were these deviations from intended intervention balanced between groups? | | | NA |  |
|  | 2.6 Was an appropriate analysis used to estimate the effect of assignment to intervention? | | | Y |  |
|  | 2.7 If N/PN/NI to 2.6: Was there potential for a substantial impact (on the result) of the failure to analyse participants in the group to which they were randomized? | | | NA |  |
|  | Risk of bias judgement | | | Low |  |
| Bias due to missing outcome data | 3.1 Were data for this outcome available for all, or nearly all, participants randomized? | | | Y |  |
|  | 3.2 If N/PN/NI to 3.1: Is there evidence that result was not biased by missing outcome data? | | | NA |  |
|  | 3.3 If N/PN to 3.2: Could missingness in the outcome depend on its true value? | | | NA |  |
|  | 3.4 If Y/PY/NI to 3.3: Is it likely that missingness in the outcome depended on its true value? | | | NA |  |
|  | Risk of bias judgement | | | Low |  |
| Bias in measurement of the outcome | 4.1 Was the method of measuring the outcome inappropriate? | | | N |  |
|  | 4.2 Could measurement or ascertainment of the outcome have differed between intervention groups? | | | N |  |
|  | 4.3 Were outcome assessors aware of the intervention received by study participants? | | | N |  |
|  | 4.4 If Y/PY/NI to 4.3: Could assessment of the outcome have been influenced by knowledge of intervention received? | | | NA |  |
|  | 4.5 If Y/PY/NI to 4.4: Is it likely that assessment of the outcome was influenced by knowledge of intervention received? | | | NA |  |
|  | Risk of bias judgement | | | Low |  |
| Bias in selection of the reported result | 5.1 Were the data that produced this result analysed in accordance with a pre-specified analysis plan that was finalized before unblinded outcome data were available for analysis? | | | Y |  |
|  | 5.2 ... multiple eligible outcome measurements (e.g. scales, definitions, time points) within the outcome domain? | | | N |  |
|  | 5.3 ... multiple eligible analyses of the data? | | | N |  |
|  | Risk of bias judgement | | | Low |  |
| Overall bias | Risk of bias judgement | | | Low |  |
| Unique ID | 3 | Study ID | Ganeshalingham et al. | | |
| Domain | Signalling question | | | Response | Comments |
| Bias arising from the randomization process | 1.1 Was the allocation sequence random? | | | Y |  |
|  | 1.2 Was the allocation sequence concealed until participants were enrolled and assigned to interventions? | | | Y |  |
|  | 1.3 Did baseline differences between intervention groups suggest a problem with the randomization process? | | | N |  |
|  | Risk of bias judgement | | | Low |  |
| Bias due to deviations from intended interventions | 2.1.Were participants aware of their assigned intervention during the trial? | | | N | The effect size was so large vs placebo there is the possibility participants would have known |
|  | 2.2.Were carers and people delivering the interventions aware of participants' assigned intervention during the trial? | | | N |  |
|  | 2.3. If Y/PY/NI to 2.1 or 2.2: Were there deviations from the intended intervention that arose because of the experimental context? | | | NA |  |
|  | 2.4 If Y/PY to 2.3: Were these deviations likely to have affected the outcome? | | | NA |  |
|  | 2.5. If Y/PY/NI to 2.4: Were these deviations from intended intervention balanced between groups? | | | NA |  |
|  | 2.6 Was an appropriate analysis used to estimate the effect of assignment to intervention? | | | Y |  |
|  | 2.7 If N/PN/NI to 2.6: Was there potential for a substantial impact (on the result) of the failure to analyse participants in the group to which they were randomized? | | | NA |  |
|  | Risk of bias judgement | | | Low |  |
| Bias due to missing outcome data | 3.1 Were data for this outcome available for all, or nearly all, participants randomized? | | | Y |  |
|  | 3.2 If N/PN/NI to 3.1: Is there evidence that result was not biased by missing outcome data? | | | NA |  |
|  | 3.3 If N/PN to 3.2: Could missingness in the outcome depend on its true value? | | | NA |  |
|  | 3.4 If Y/PY/NI to 3.3: Is it likely that missingness in the outcome depended on its true value? | | | NA |  |
|  | Risk of bias judgement | | | Low |  |
| Bias in measurement of the outcome | 4.1 Was the method of measuring the outcome inappropriate? | | | N |  |
|  | 4.2 Could measurement or ascertainment of the outcome have differed between intervention groups? | | | N |  |
|  | 4.3 Were outcome assessors aware of the intervention received by study participants? | | | N |  |
|  | 4.4 If Y/PY/NI to 4.3: Could assessment of the outcome have been influenced by knowledge of intervention received? | | | NA |  |
|  | 4.5 If Y/PY/NI to 4.4: Is it likely that assessment of the outcome was influenced by knowledge of intervention received? | | | NA |  |
|  | Risk of bias judgement | | | Low |  |
| Bias in selection of the reported result | 5.1 Were the data that produced this result analysed in accordance with a pre-specified analysis plan that was finalized before unblinded outcome data were available for analysis? | | | Y |  |
|  | 5.2 ... multiple eligible outcome measurements (e.g. scales, definitions, time points) within the outcome domain? | | | N |  |
|  | 5.3 ... multiple eligible analyses of the data? | | | N |  |
|  | Risk of bias judgement | | | Low |  |
| Overall bias | Risk of bias judgement | | | Low |  |

**Supplementary Table 4**: Justification of certainty assessment for continuous outcomes

| Outcome | Downgrade domain(s) | Rationale for downgrade | Final certainty |
| --- | --- | --- | --- |
| Body weight (kg) | None | Pooled effect large and clinically meaningful; 95% CI and 95% prediction interval both excluded the null. | High |
| BMI (kg/m²) | None | Effect was clinically meaningful; confidence and prediction intervals excluded the null; between-study variability did not threaten consistency of effect. | High |
| HbA1c (%) | None | Narrow confidence and prediction intervals excluding the null; minimal between-study heterogeneity; findings consistent across trials. | High |
| Fasting glucose (mmol/L) | Inconsistency / Imprecision (–1) | Although the pooled estimate and 95% CI indicated benefit, the 95% prediction interval crossed the null, suggesting that benefit may not be consistent across all plausible settings. | Moderate |
| Systolic blood pressure (mmHg) | Imprecision (–1) | Confidence and prediction intervals crossed the null, indicating substantial uncertainty in the magnitude and direction of effect. | Low |
| Diastolic blood pressure (mmHg) | Imprecision (–1) | Estimate showed no clear effect; both confidence and prediction intervals crossed the null, limiting confidence. | Low |
| Fasting insulin (mU/L) | Imprecision (–1); Inconsistency (–1) | Wide confidence and prediction intervals crossing the null, combined with moderate between-study heterogeneity. | Very low |
| Fasting triglycerides (mmol/L) | Imprecision (–1); Inconsistency (–1) | Confidence and prediction intervals crossed the null; effects were small and variable across studies, with limited precision. | Very low |
| HDL cholesterol (mmol/L) | Imprecision (–1); Inconsistency (–1) | Confidence and prediction intervals crossed the null, with small and inconsistent effects across studies. | Very low |
| Vomiting | None | Risk ratio and prediction interval excluded the null, indicating a consistent increased risk across studies. | High |
| Abdominal pain | None | Risk ratio and prediction interval excluded the null, suggesting a consistent increased risk across studies. | High |
| Constipation | Inconsistency / Imprecision (–1) | Although the pooled estimate suggested increased risk, the prediction interval crossed the null and between-study heterogeneity was present. | Moderate |
| Nausea | Imprecision (–1) | Confidence and prediction intervals crossed the null, resulting in uncertainty regarding the magnitude and consistency of increased risk. | Low |
| Diarrhea | Imprecision (–1) | Wide confidence and prediction intervals crossing the null, combined with moderate heterogeneity, limited confidence in a consistent. | Low |
| Serious adverse event (any) | Imprecision (–1) | Low event rates and confidence and prediction intervals crossing the null limited precision and confidence in an effect. | Low |

All outcomes started with a high degree of certainty due to the study design.

**Supplementary Table 5**: Outcomes not included in the meta-analysis

|  | Outcome | Authors | Study level treatment effect (95% CI) | Direction of effect vs placebo | Summary if k=2 |
| --- | --- | --- | --- | --- | --- |
| Anthropometric & Body Composition | Bodyfat (%) | Siskind et al. | -2.86  (-3.68; -2.04) | Decrease  (Significant) | Consistent decrease |
|  |  | Sass et al. | -6.10  (-11.18; -1.02) | Decrease  (Significant) |  |
|  | Bodyweight (%) | Siskind et al. | -13.46  (-16.00; -10.92) | Decrease  (Significant) | - |
|  | Bone mineral (kg) | Siskind et al. | -0.03  (-0.11; 0.05) | Decrease  (NS) | - |
|  | Fat mass (kg) | Siskind et al. | -8.93  (-13.14; -4.72) | Decrease  (Significant) | - |
|  | Fat mass lean mass ratio | Siskind et al. | -0.10  (-0.21; 0.01) | Decrease  (NS) | - |
|  | Hip circumference (cm) | Ganeshalingam et al. | -4.25  (-6.32; -2.18) | Decrease  (Significant) | - |
|  | Lean mass (kg) | Siskind et al. | -3.95  (-6.09; -1.81) | Decrease  (Significant) | - |
|  | Visceral fat (kg) | Siskind et al. | -0.72  (-1.23; -0.21) | Decrease  (Significant) | Consistent decrease |
|  |  | Sass et al. | -0.27  (-0.79; 0.25) | Decrease  (NS) |  |
|  | Waist circumference | Sass et al. | -7.00  (-10.88; -3.12) | Decrease  (Significant) | Consistent decrease |
|  |  | Ganeshalingam et al. | -6.19  (-8.86; -3.52) | Decrease  (Significant) |  |
|  | Waist-hip ratio | Siskind et al. | 0.00  (-0.03; 0.03) | No change | - |
| Cardiometabolic & Glycaemic | C-peptide (ng/mL) | Sass et al. | 0.10  (-0.45; 0.65) | Increase  (NS) | Inconsistent effect |
|  |  | Ganeshalingam et al. | -0.36  (-0.82; 0.10) | Decrease  (NS) |  |
|  | Insulin resistance (HOMA) | Siskind et al. | -1.21  (-3.86; 1.44) | Decrease  (NS) | Consistent decrease |
|  |  | Sass et al. | -0.01  (-0.5; 0.48) | Decrease  (NS) |  |
|  | FCI | Siskind et al. | 1.93  (-10.79; 14.65) | Increase  (NS) | - |
| Lipids & Vascular Markers | LDL | Sass et al. | -1.50  (-16.07; 13.07) | Decrease  (NS) | Inconsistent effect |
|  |  | Ganeshalingam et al. | 0.00  (-6.43; 6.43) | No change |  |
|  | Total cholesterol | Sass et al. | -1.20  (-18.68; 16.28) | Decrease  (NS) | Inconsistent effect |
|  |  | Ganeshalingam et al. | 12.74  (-13.34; 38.82) | Increase  (NS) |  |
|  | Triglycerides | Sass et al. | -8.90  (-42.88; 25.08) | Decrease  (NS) | Consistent decrease |
|  |  | Ganeshalingam et al. | -29.20  (-69.27; 10.87) | Decrease  (NS) |  |
|  | Heart rate (bpm) | Siskind et al. | -3.64  (-12.16; 4.88) | Decrease  (NS) | Consistent decrease |
|  |  | Sass et al. | -1.50  (-7.26; 4.26) | Decrease  (NS) |  |
| Liver & Renal Biomarkers | Alkaline phosphatase (U/L) | Sass et al. | -14.1  (-22.36; -5.84) | Decrease  (Significant) | - |
|  | ALT (U/L) | Sass et al. | -2.57  (-15.27; 10.13) | Decrease  (NS) | - |
|  | AST (U/L) | Sass et al. | 2.45  (-2.12; 7.02) | Decrease  (NS) | - |
|  | Amylase (U/L) | Sass et al. | -1.1  (-6.52; 4.32) | Decrease  (NS) | Inconsistent effect |
|  |  | Ganeshalingam et al. | 4.98  (1.57; 8.39) | Increase (Significant) |  |
|  | FIB-4 | Siskind et al. | -0.02  (-0.12; 0.08) | Decrease  (NS) | Inconsistent effect |
|  |  | Sass et al. | 0.10  (0.00; 0.20) | Increase  (NS) |  |
|  | Creatinine (mg/dL) | Ganeshalingam et al. | 0.04  (0.01; 0.07) | Increase (Significant) | - |
|  | CRP (mg/dL) | Ganeshalingam et al. | -0.11  (-0.43; 0.21) | Decrease  (NS) | - |
|  | Urate (mg/dL) | Ganeshalingam et al. | -0.50  (-1.03; 0.03) | Decrease  (NS) | - |
|  | Hemoglobin (g/dL) | Ganeshalingam et al. | 0.10  (-0.11; 0.31) | Increase  (NS) | - |
|  | NAFLD Fibrosis Score | Siskind et al. | -0.37  (-0.90; 0.16) | Decrease  (NS) | - |
| Symptom Severity | PANSS general psychopathology | Siskind et al. | 1.65  (-2.26; 5.56) | Increase  (NS) | - |
|  | PANSS negative | Siskind et al. | -0.92  (-2.97; 1.13) | Decrease  (NS) | - |
|  | PANSS positive | Siskind et al. | -0.62  (-2.82; 1.58) | Decrease  (NS) | - |
|  | PANSS total | Siskind et al. | 0.20  (-5.73; 6.13) | Increase  (NS) | - |
|  | PANSS-6 | Sass et al. | 0.71  (-2.1; 3.52) | Increase  (NS) | - |
|  | CGI-S | Sass et al. | 0.19  (-0.31; 0.69) | Increase  (NS) | - |
| Cognition & Functioning | BACS animals | Siskind et al. | -0.13  (-0.75; 0.49) | Decrease  (NS) | - |
|  | BACS digital sequencing task | Siskind et al. | 0.16  (-0.52; 0.84) | Increase  (NS) | - |
|  | BACS F words | Siskind et al. | -0.22  (-0.81; 0.37) | Decrease  (NS) | - |
|  | BACS S words | Siskind et al. | 0.25  (-0.21; 0.71) | Increase  (NS) | - |
|  | BACS symbol coding task | Siskind et al. | -0.19  (-0.56; 0.18) | Decrease  (NS) | - |
|  | BACS verbal memory learning | Siskind et al. | 0.62  (0.09; 1.15) | Increase (Significant) | - |
|  | Trail making test A (errors) | Siskind et al. | -0.44  (-1.22; 0.34) | Decrease  (NS) | - |
|  | Trail making test A (time) | Siskind et al. | 0.38  (-0.30; 1.06) | Increase  (NS) | - |
|  | Trail making test B (errors) | Siskind et al. | 0.12  (-0.50; 0.74) | Increase  (NS) | - |
|  | Trail making test B (time) | Siskind et al. | -0.23  (-0.70; 0.24) | Decrease  (NS) | - |
|  | TOPF | Siskind et al. | 0.57  (-4.06; 5.20) | Increase  (NS) | - |
|  | GAPD | Sass et al. | 0.24  (-0.49; 0.97) | Decrease  (NS) | - |
| Substance Use, Quality of Life, and lifestyle | Alcohol intake (AUDIT) | Siskind et al. | -0.81  (-2.34; 0.72) | Decrease  (NS) | Inconsistent effect |
|  |  | Sass et al. | 0.57  (-0.68; 1.82) | Increase  (NS) |  |
|  | Alcohol intake  (AUDIT-C) | Sass et al. | -0.16  (-0.89; 0.57) | Decrease  (NS) | - |
|  | DUDIT | Sass et al. | 0.41  (-0.56; 1.38) | Increase  (NS) | - |
|  | FTND | Sass et al. | -1.36  (-2.26; -0.46) | Decrease  (Significant) | - |
|  | SQLS adverse effects | Sass et al. | -0.16  (-0.85; 0.53) | Decrease  (NS) | - |
|  | SQLS motivation | Sass et al. | -0.33  (-1.08; 0.42) | Decrease  (NS) | - |
|  | SQLS psychosocial | Sass et al. | -0.43  (-1.69; 0.83) | Decrease  (NS) | - |
|  | MVPA (SIMPAQ) | Siskind et al. | -6.78  (-203.92; 190.36) | Decrease  (NS) | - |
|  | Sedentary behaviour (SIMPAQ) | Siskind et al. | -36.30  (-171.61; 99.01) | Decrease  (NS) | - |
| Psychotropic Drug Pharmacokinetics | Clozapine levels | Siskind et al. | -39.66  (-201.47; 122.15) | Decrease  (NS) | - |
|  | Clozapine/norclozapine ratio | Siskind et al. | 0.85  (-0.73; 2.43) | Increase  (NS) | - |
|  | Norclozapine | Siskind et al. | -33.87  (-116.29; 48.55) | Decrease  (NS) | - |

ALT = alanine aminotransferase; AST = aspartate aminotransferase; CGI-S = Clinical Global Impression – Severity; DUDIT = Drug Use Disorders Identification Test; FCI = Framingham Cardiovascular Risk Index; FTND = Fagerström Test for Nicotine Dependence; GAPD = Global Assessment of Psychosocial Disability; NS=non-significant

**Supplementary Table 6**: Justification of certainty assessment for continuous outcomes

| Adverse event | Starting certainty | Downgrade domain(s) | Rationale for downgrade | Final credibility |
| --- | --- | --- | --- | --- |
| Vomiting | High | None | Pooled risk ratio and 95% prediction interval excluded the null, indicating a consistent increased risk across studies with minimal heterogeneity; findings align with known GLP-1 receptor agonist class effects. | High |
| Abdominal pain | High | None | Pooled risk ratio and prediction interval excluded the null, suggesting a consistent increased risk across studies despite modest imprecision. | High |
| Constipation | High | Inconsistency / Imprecision (–1) | Although the pooled estimate suggested increased risk, the prediction interval crossed the null and between-study heterogeneity was present, indicating that effects may vary across clinical settings. | Moderate |
| Nausea | High | Imprecision (–1) | Confidence and prediction intervals crossed the null, resulting in uncertainty regarding the magnitude and consistency of increased risk despite biological plausibility. | Low |
| Diarrhea | High | Imprecision (–1) | Wide confidence and prediction intervals crossing the null, combined with moderate heterogeneity, limited confidence in a consistent effect across studies. | Low |
| Serious adverse event (any) | High | Imprecision (–1) | Low event rates and confidence and prediction intervals crossing the null limited precision and confidence in an effect on serious adverse events. | Low |

**Supplementary Table 7**: Adverse event outcomes not included in the meta-analysis

| **Category** | **Adverse event** | **Authors** | **Risk ratio (RR)** | **Direction vs control** | **Summary if k=2** |
| --- | --- | --- | --- | --- | --- |
| Any / overall | Any adverse event | Ganeshalingam et al. | 1.00 | No difference | - |
|  | Any AE | Siskind et al. | 1.36 | Increase | - |
|  | Any AE leading to discontinuation | Sass et al. | 1.03 | Increase | - |
| Gastrointestinal | Abdominal discomfort | Siskind et al. | 1.07 | Increase | - |
|  | Acid reflux | Siskind et al. | 3.19 | Increase | - |
|  | Dyspepsia | Ganeshalingam et al. | 1.92 | Increase | - |
|  | Flatulence | Sass et al. | 3.08 | Increase | Inconsistent |
|  | Flatulence | Ganeshalingam et al. | 1.88 | Increase |  |
| Neurological / constitutional | Dizziness | Siskind et al. | 3.20 | Increase | Inconsistent |
|  | Dizziness | Sass et al. | 1.17 | Increase |  |
|  | Fatigue | Siskind et al. | 1.07 | Increase | Inconsistent |
|  | Fatigue | Sass et al. | 1.03 | Increase |  |
| Psychiatric | Suicidal ideation | Siskind et al. | 3.19 | Increase | - |
|  | Self-harm | Sass et al. | 1.54 | Increase | - |
| Injection site | Injection site bruising | Siskind et al. | 1.33 | Increase | - |
|  | Injection site reaction | Sass et al. | 2.06 | Increase | - |
| Other / systemic | Heart failure | Sass et al. | 3.08 | Increase | - |
|  | Pneumonia | Sass et al. | 0.21 | Decrease | - |
|  | Sudden death | Sass et al. | 3.08 | Increase | - |
